# Self-directed exploration provides a *Ncs1*-dependent learning bonus

**DOI:** 10.1038/srep17697

**Published:** 2015-12-07

**Authors:** Ho-Suk Mun, Bechara J. Saab, Enoch Ng, Alexander McGirr, Tatiana V. Lipina, Yoichi Gondo, John Georgiou, John C. Roder

**Affiliations:** 1 Lunenfeld-Tanenbaum Research Institute, Mount Sinai Hospital, Toronto, ON, M5G 1X5, Canada; 2 Department of Molecular Genetics, University of Toronto, Toronto, ON, M5S 1A8, Canada; 3 Preclinical Laboratory for Translational Research into Affective Disorders, University of Zurich Hospital for Psychiatry, August-Forel-Str 7, CH-8008, Zurich, Switzerland; 4 Neuroscience Center Zurich, Zurich, CH-8057, Switzerland; 5 Institute of Medical Science, University of Toronto, Toronto, ON, M5S 1A8, Canada; 6 Department of Psychiatry, University of British Columbia, Vancouver, BC, V6T 2A1, Canada; 7 Federal State Budgetary Scientific Institution, Scientific Research Institute of Physiology and Basic Medicine, Novosibirsk, 630117, Russia; 8 Mutagenesis and Genomics Team, RIKEN BioResource Center, Tsukuba 305-0074, Japan; 9 Department of Physiology, University of Toronto, Toronto, ON, M5S 1A8, Canada

## Abstract

Understanding the mechanisms of memory formation is fundamental to establishing optimal educational practices and restoring cognitive function in brain disease. Here, we show for the first time in a non-primate species, that spatial learning receives a special bonus from self-directed exploration. In contrast, when exploration is escape-oriented, or when the full repertoire of exploratory behaviors is reduced, no learning bonus occurs. These findings permitted the first molecular and cellular examinations into the coupling of exploration to learning. We found elevated expression of neuronal calcium sensor 1 (*Ncs1*) and dopamine type-2 receptors upon self-directed exploration, in concert with increased neuronal activity in the hippocampal dentate gyrus and area CA3, as well as the nucleus accumbens. We probed further into the learning bonus by developing a point mutant mouse (*Ncs1*^P144S/P144S^) harboring a destabilized NCS-1 protein, and found this line lacked the equivalent self-directed exploration learning bonus. Acute knock-down of *Ncs1* in the hippocampus also decoupled exploration from efficient learning. These results are potentially relevant for augmenting learning and memory in health and disease, and provide the basis for further molecular and circuit analyses in this direction.

Understanding the underlying mechanisms of learning and memory (**LM**) is of great importance for educational practices and restoring cognitive function in human aging and brain disorders. Upstream of LM are the processes by which we explore, gather and organize the raw information from the environment on which memories are built. Here we investigated the molecular and behavioral mechanisms that mediate self-directed exploration and its associated benefits for spatial LM.

In psychological research and behavioral research, “exploration” widely refers to all activities directed at increasing information intake from the environment^1^. Exploration has important implications for survival, and can give rise to sources of food, safety, and reproduction^2^,^3^, which may explain the clear evolutionarily conserved reward intrinsic to investigation of novel stimuli^4^,^5^.

In humans, self-directed “volitional” exploration of an object and its location results in improved recognition performance, and this benefit is linked to a brain network centered on the hippocampus^6^. Similarly in rats, the experimentally manipulated motivation to explore objects differentially recruits specific sub-regions of the hippocampus^7^, and our previous research in the mouse revealed the importance of the dentate gyrus (**DG**) in driving exploratory behavior of safe, novel space (**SNS**)^8^.

The type, extent and underlying motivation for exploration varies with environmental factors, such as novelty, degree of stress and potential for food or mating; all of which also affect LM performance^6^,^9^,^10^,^11^,^12^. In particular, safe non-threatening environments improve academic performance^12^, while mild stress can impair memory formation in humans^11^. Here, we experimentally manipulated the level of fear during self-directed exploration by employing different lighting and novelty conditions in order to examine the underlying basis for the effects that the motivation to explore has on LM.

We have previously shown that over-expression of neuronal calcium sensor1 (***Ncs1***) in the murine DG increases exploratory behavior and spatial LM^8^. NCS-1 is a member of the neuronal calcium sensor protein superfamily^13^ that regulates presynaptic Ca^2+^ influx in flies^14^ and long-term synaptic plasticity in rodents^8^,^15^,^16^. NCS-1 is also key for LM in *C. elegans*^17^.

Yet, NCS-1 and its binding partners may further facilitate LM in concert with motivation and reward circuitry to seek out additional unique environmental inputs, or to more efficiently encode information about the surrounding environment. Indeed, allelic variation in *Ncs1* is implicated in addictive behaviors^18^, characterized by pathological motivation and reward. Overexpression of NCS-1 in the hippocampal DG promotes curiosity and the acquisition of spatial memory, likely through regulating surface expression of the dopamine D2 receptor (**D2DR;** ***Drd2***)^8^, a binding partner of NCS-1^8^,^19^. Chronic cocaine exposure reduces D2DR-modulated Ca^2+^ influx and activity of the adenylate cyclase-calcineurin pathway in the nucleus accumbens (**NAc**)^20^,^21^. *Ncs1* knock-out mice exhibit impaired exploratory behavior, together with deficits in non-aversive long-term memory^22^. Thus, in addition to the established role of hippocampal NCS-1 in exploratory drive and LM, extra-hippocampal NCS-1 may govern the degree of intrinsic reward associated with exploration.

We provide data from mice showing that volitional vertical exploration, quantified by rearing, correlates with better spatial learning in an object discrimination task involving object displacement, and that experimentally enhancing and preventing this form of exploration, in turn, enhances and prevents spatial learning, respectively. Furthermore, we demonstrate, through multiple independent approaches, that NCS-1 in the brain, and specifically the DG, is a factor for intrinsically motivated exploratory behavior and the associated learning bonus. Our experiments show that exploration up-regulates expression of unique genes including *Ncs1* and *Drd2*, and that knock-down of NCS-1 levels by siRNA or by point mutation reduces exploration, causing deficient spatial memory. Thus we demonstrate that NCS-1 is not only essential for cognitive capacity, but also regulates genes that are related to intrinsically motivated exploration, something that could be considered akin to curiosity.

## Results

**Figure 1:**
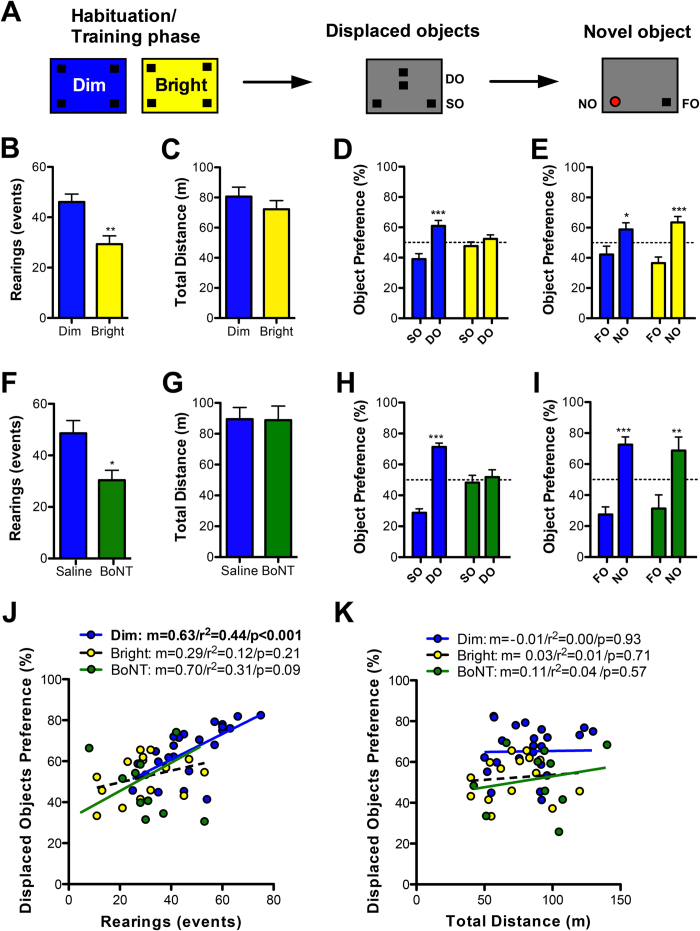
Exploratory rearing promotes spatial learning. (**A**) Diagram showing object recognition testing procedure. (**B**–**E**) Relationship between exploratory rearing and spatial memory in the object recognition test. Mice were trained in an arena with four objects and under dim (n = 14) or bright (n = 15) lighting while rearing (**B**) and horizontal travel distance (**C**) were recorded. (**D**) Later, preference towards two displaced objects (DO) over stationary objects (SO) was evaluated. (**E**) Ability to recognize a novel object (NO) over a familiar object (FO) was evaluated. (**F**–**I**) Relationship between exploratory rearing and spatial memory in the object recognition test for mice injected with saline or botulinum toxin A (BoNT) into hindlimb muscle, the latter of which caused focal hindlimb paresis. Rearings (**F**) and horizontal travel distance (**G**) were recorded during object habituation and subsequent testing for DO preference (**H**) and NO preference (**I**). (**J**) Plot of rearing data (**B**,**F**) against DO memory (**D**,**H**) (**K**) Plot of travel distance data (**C**,**G**) against DO memory (**D**,**H**). Data are expressed as mean ± SEM. Pearson correlation (r^2^) and slope (m) were determined. *P < 0.05, **P < 0.01, ***P < 0.001. See also Supplementary Figure S1 and Table S1.

To define precise links between exploration and learning, and then study the potential role of NCS-1 in these two processes, we first searched for a behavioral assay that met two specific criteria: (1) it must allow for the quantification of both exploration and learning and the correlation of the two measures, and (2) it must provide a way to manipulate the level of exploration. Since the object recognition test satisfies the first criteria (Fig. 1A), and modulating the level of perceived threat by adjusting the brightness of arena lighting also affects the level of exploration (Fig. 1B,C; Supplementary Figure S1A,B), this task became our primary measure of exploration/learning.

### High exploration and enhanced spatial learning in novel, dimly-lit environments

We studied whether lighting conditions (Dim vs. Bright) alter various measures of exploratory behavior in an open field containing novel objects (Fig. 1A). Dimly-lit novel environments specifically increased two forms of self-directed exploration, namely vertical exploration in the form of rearing (on hind-limb) frequency and horizontal exploration in the form of path length travelled in the center of the arena (Fig. 1B,C; Supplementary Figure S1A,B). Dimly-lit novel environments did not affect overall ambulation (Supplementary Figure S1C), grooming (Supplementary Figure S1D) or object bias (Supplementary Figure S1I,L). Therefore, we interchangeably refer to the dimly-lit condition as a high-exploration environment and the brightly-lit condition as a low-exploration environment.

Rearing in SNS allows animals to make use of a superior vantage point^23^, from which they can see farther, sniff alternative air currents and potentially obtain novel auditory information. If rearing is an exploratory strategy that improves the acquisition of spatial information, it may be associated with improved spatial learning. Alternatively, rearing could coincide with a brain state independently primed for efficient learning.

After training in either high- or low-exploration environments, we tested performance for displaced and novel object recognition memory, the former being more difficult and hippocampal-dependent. Interestingly, only the group trained in the high-exploration environment showed a preference for the displaced objects, implying a superior spatial map of the environment relative to the low-exploration group (Fig. 1D). In contrast, both groups of mice demonstrated a preference for the novel object in phase three of the task (Fig. 1E). These results suggest a special role for the hippocampus in coupling exploration to efficient learning.

While a high-exploration environment favors rearing, a dimly-lit environment is intrinsically less threatening and therefore may improve displaced object memory in a manner not contingent on exploration. We thus sought to explore the contingent relationship between rearing and displaced object memory by selectively limiting rearing by way of focal partial hind-limb paresis induced via botulinum neurotoxin type A (**BoNT**; XEOMIN) injection to the quadricep^24^. We established an optimal dose of 0.1 units that impaired rearing but not horizontal ambulation or other behaviors, including time spent in the center of the arena (Fig. 1F,G; Supplementary Figure S1E–H,L). Despite being trained in a non-threatening dimly-lit high-exploration environment, and otherwise performing behaviorally the same as controls, BoNT-treated mice were impaired in memory for displaced object location (Fig. 1H). Novel object recognition on the other hand was unaffected (Fig. 1I), showing the general ability to learn remained intact. We therefore conclude that the act of rearing, and the ability to explore at will, is essential for efficient learning

To further evaluate the role of exploratory rearing in learning, we examined the correlation (Fig. 1J; Supplementary Table S1) between rearing (Fig. 1B,F) during the training phase and displaced object preference (Fig. 1D,H). We found that subjects trained in dim lighting that had the most rearing events also showed the highest preference for displaced objects (Fig. 1J; blue dots). The same relationship was not observed for mice trained in the bright environment (Fig. 1J; yellow dots), indicating that the level of exploration *per se* may not be the critical factor that elicits a learning bonus, but instead requires rearing combined with the appropriate motivation to explore. This is further supported by the fact that BoNT-treated mice (Fig. 1J; green dots), that were trained in dim lighting, showed a leftward shifted range of data but with slope similar to that of untreated explorers, and relatively high correlation coefficient value (not significant; p = 0.09). We found no correlation between horizontal movement and spatial memory in any of the three groups (Fig. 1K; Supplementary Table S1).

In the event that mice trained under low-exploration conditions performed poorly on the object displacement task as a result of interpreting the arena during the object displacement phase (always dimly-lit) as different from the arena during the habituation phase (brightly-lit for the low-exploration group), we compared the level of rearing in the object displacement phase for the two groups of animals as a measure of novelty, but found no differences (Supplementary Figure S1J).

### Exploration of SNS activates the DG, CA3, NAc and expression of *Ncs1* and *Drd2*

**Figure 2:**
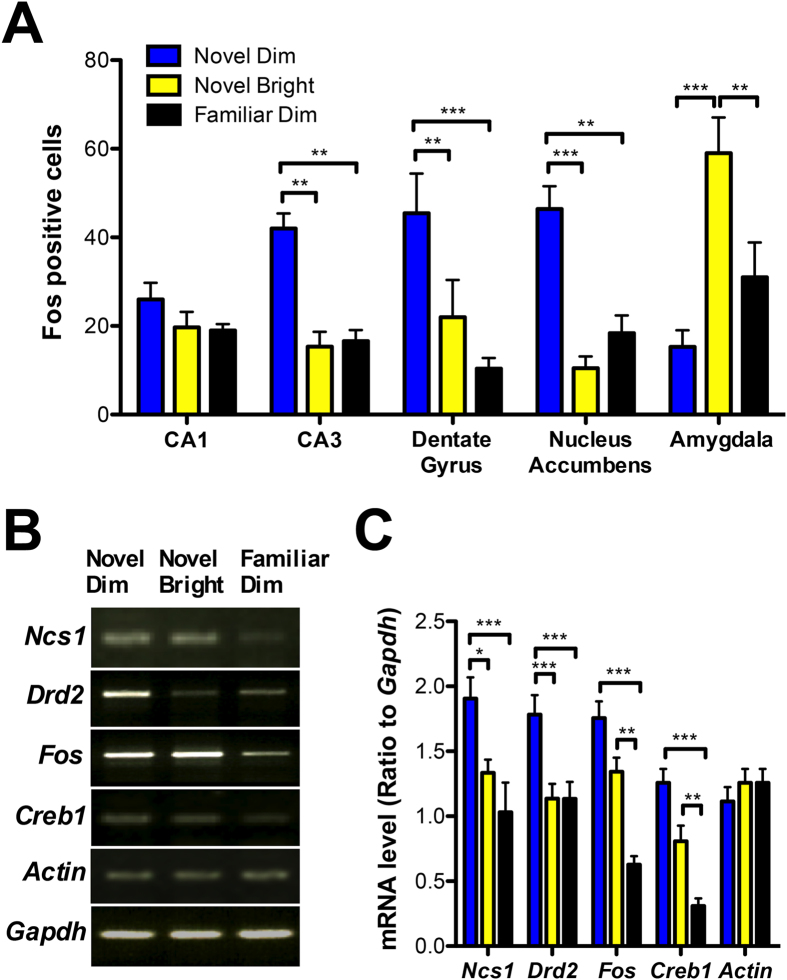
Novel dim environments that enhance rearing induce c-Fos and elevate *Ncs1* and *Drd2* expression. (**A**) Counts of c-Fos-positive cells in brain regions at 90 minutes post-exposure to a novel dim, novel bright, or familiar dim environment. Three sections per mouse, n = 3 per group. (**B**,**C**) Comparison of various mRNA transcripts in the hippocampus 30 minutes following exposure to testing environments. (**B**) Representative images of electrophoresed RT-PCR amplicons for indicated mRNA. (**C**) Ratio of *Ncs1*, *Drd2*, *Fos, Creb1*, and *Actin* mRNA levels normalized to *Gapdh* (n = 3 per group, triplicate). Data are expressed as mean ± SEM. *P < 0.05, **P < 0.01, ***P < 0.001. See also Supplementary Figure S2.

To gain insight into the neural and molecular substrates responsible for differences in exploratory behavior and the efficiency to learn in low- versus high-exploration environments, we examined the expression of the immediate early gene *Fos* as a marker of neuronal activity^25^,^26^ during the acquisition phase of the object recognition paradigm. We focused on the hippocampus, due to its suggested role in spatial navigation, exploration and novelty detection^8^,^27^,^28^; the NAc as it is involved in processing motivational relevance of environmental stimuli^29^; and the basolateral amygdala as a fear center^30^. We found SNS, high-exploration environments, were associated with more c-Fos-positive cells in the DG, hippocampal subregion CA3 and the NAc (Fig. 2A; Supplementary Figure S2). Novel bright environments, in contrast, only led to pronounced increases in amygdalar c-Fos-positive cells (Fig. 2A; Supplementary Figure S2).

Given the prior work that links D2DR and NCS-1 to novelty-driven exploration and spatial learning^8^, we examined whether changes in *Drd2* and *Ncs1* expression are modulated by exposure to environments that vary in lighting and novelty. Quantitative polymerase chain reaction (**PCR**) revealed *Ncs1* and *Drd2* are specifically upregulated following exploration in SNS, in comparison to exploration in either novel bright or familiar dim environments (Fig. 2B,C).

Two additional transcripts were uniquely modified between conditions. The known memory regulator cAMP Response Element-Binding (*Creb*) and *Fos* both increased as a function of novelty, independent of the lighting or level of exploration (Fig. 2B,C), confirming our immunohistofluorescence data (Fig. 2A; Supplementary Figure S2). This result may suggest that novelty-induced exploration activates the hippocampus and causes an elevation in genes known to be involved in synaptic plasticity and memory encoding. In contrast, *Ncs1* and *Drd2* were elevated only when novelty was coupled with a safe environment and high levels of rearing, an intriguing result that led us to wonder whether NCS-1 is required for the learning bonus provided by exploration.

### NCS-1 is required for exploration-enhanced learning

**Figure 3:**
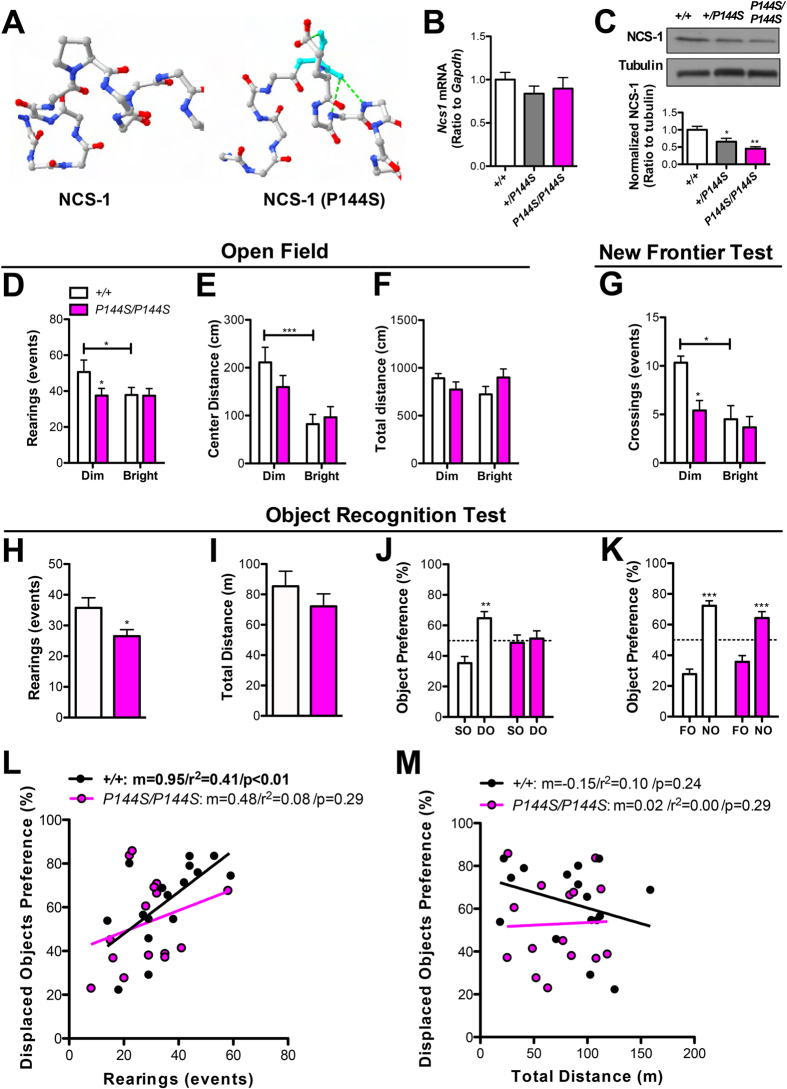
*Ncs1*^*P144S/P144S*^ mice show reduced exploration and impaired spatial memory. (**A**) Models of wild-type (left) and P144S (right) NCS-1 protein. (**B**) *Ncs1* mRNA levels between genotypes. (**C**) Representative immunoblot (upper) of NCS-1 using tubulin as loading control with densitometric quantification (lower). (**D**–**G**) Exploratory activity for *Ncs1*^*P144S/P144S*^ mice (n = 12 per group) in the open field (**D**–**F**) and New Frontier tests (**G**) were conducted under dim or bright lighting and the following measures were recorded: rearing (**D**), ambulation in the center of (**E**) or over the whole arena (**F**), and crossing events (**G**). (**H**–**K**) Object recognition testing showing rearings (**H**) and travelled distance (**I**) during training phase, and preference for displaced (**J**) or novel objects (**K**). (**L**) Correlation plot for displaced object preference as a function of exploratory rearing. (**M**) Correlation plot between horizontal movement and displaced object preference. *Ncs1*^*P144S/P144S*^, n = 16; +/+, n = 15; Data are expressed as mean ± SEM. Pearson correlation (r^2^) and slope (m) are shown. * p < 0.05, ** p < 0.01. DO; displaced objects, SO; stationary objects, NO; novel object, FO; familiar object. See also Supplementary Figures S3,S4, and Table S1.

To test the molecular relationship between NCS-1 and exploration, we generated a mouse mutant of the protein by screening the RIKEN ENU-mutagenized genomic DNA archive. One resulting mouse, *Ncs1*^*P144S/P144S*^ harbored a point mutation that introduces a third degree of freedom of rotation at residue 144 and potential for at least two additional hydrogen bonds that could interfere with proper protein folding and/or give rise to protein instability (Fig. 3A; Supplementary Figure S3A,B; see also Supplementary Figures S3,S4 and Supplemental Methods). Quantitative PCR revealed no differences in whole brain *Ncs1* mRNA between P144S and wild-type genotypes (Fig. 3B), however, immunoblots revealed a gene dose-dependent reduction in NCS-1 protein (Fig. 3C). The reduction in protein but not mRNA levels is consistent with the in silico predicted destabilization of protein structure (Fig. 3A). In the event our immunoblots were confounded by an antibody-affinity effect, we confirmed the reduction of NCS-1 protein levels in the hippocampus by using a second antibody (Supplementary Figure S3C,D).

*Ncs1*^*P144S/P144S*^ mice were of normal weight (Supplementary Figure S3E) and showed no abnormalities in measures of general physiology, such as walking and grooming (Supplementary Figure S3F) in the open field test and motor function in the rotarod test (Supplementary Figure S3G). We also examined endophenotypes of mental illness, such as anxiety (Supplementary Figure S3H–J), depression and addiction (Supplementary Figure S3K,L), but found no significant differences. However, *Ncs1*^*P144S/P144S*^ mice reared less frequently than their littermate controls in the open field (Fig. 3D,H; Supplementary Figure S4A), and spent less time in the center of the novel arenas (Fig. 3E) despite normal horizontal activity and its habituation (Fig. 3F,I; Supplementary Figure S4B).

In the New Frontier task^8^, *Ncs1*^*P144S/P144S*^ subjects explored from their home cages, but showed fewer total visits to the novel frontiers (Fig. 3G), and in the hole-board task, demonstrated fewer hole-pokes compared to controls (Supplementary Figure S4C). Interestingly, in both cases, there were no genotypic differences in the exploratory behavior when tests were performed in a threatening, low-exploration environment (Fig. 3D–G; Supplementary Figure S4A,B), suggesting NCS-1 is specifically required for the type of intrinsically rewarding exploration that we find is coupled to efficient learning.

We proceeded to examine whether a link between exploratory rearing and spatial memory exists in our genetic model. The NCS-1 mutants reared less without changing overall ambulation during object recognition training and showed poorer displaced object memory and intact novel object recognition (Fig. 3H–K). A correlation analysis revealed significant association between rearing activity and spatial memory in *Ncs1*^*+/+*^but not *Ncs1*^*P144S/P144S*^ mice (Fig. 3L; Supplementary Table S1), suggesting NCS-1 is critical for the learning bonus afforded by self-directed exploration. In contrast, no correlation was found between horizontal movement and spatial memory, irrespective of genotype (Fig. 3M; Supplementary Table S1).

**Figure 4:**
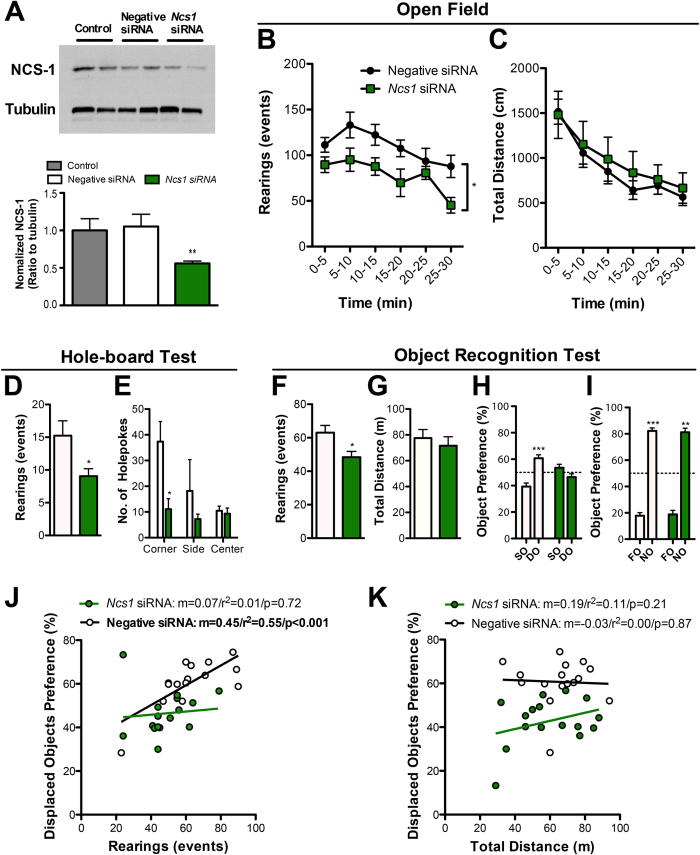
*Ncs1* knock-down in dentate gyrus (DG) reduces exploration. (**A**) Representative immunoblot for hippocampal NCS-1 and tubulin three days after the DG was infused with negative siRNA or siRNA against *Ncs1*, as compared to untreated control mice on the left with densitometric analysis (normalized to tubulin) showing that *Ncs1* siRNA reduced NCS-1 protein level (two representative samples per group). Mice were tested in the open field (**B**,**C**) and hole-board test (**D**,**E**) under dim lighting and measured for rearing (**B**,**D**), distance (**C**) and holepoke exploration (**E**). (**F**–**I**) Relationship between exploratory rearing and spatial memory in the object recognition test for *Ncs1* siRNA- and negative siRNA-treated mice. Reduced rearing (**F**) and normal travel distance (**G**) during object habituation and subsequent testing for displaced object preference (DO) over stationary objects (SO) (**H**). (**I**) Intact novel object (NO) over former object (FO) preference in all siRNA-treated mice. (**J**) Plot of exploratory rearing data (**F**) against displaced object memory (**H**). (**K**) Correlation plot between horizontal movement and displaced object preference. Negative siRNA, n = 13; *Ncs1* siRNA, n = 11. Data are expressed as mean ± SEM. Pearson correlation (r^2^) and slope (m) are shown. *p < 0.05, **p < 0.01. See also Supplementary Figure S5 and Table S1.

To determine whether NCS-1 in the DG, specifically, is required for exploration, we proceeded to acutely reduce DG NCS-1 using siRNA. We initially screened a set of siRNAs targeting *Ncs1* in mouse neuroblastoma Neuro2A cells and found one siRNA (Freq 3) reduced NCS-1 protein levels relative to the negative siRNA (Supplementary Figure S5A,B). Infusion of Freq 3 siRNA directly into the DG of cannulated wild-type C57BL/6J mice reduced the total amount of basal NCS-1 protein in the hippocampus to about half (Fig. 4A). While the manipulation did not affect walking or grooming (Supplementary Figure S5C), rearing was reduced during the training phase of the object recognition task (Fig. 4B,C). Like the low-explorers and *Ncs1* P144S point mutants, specific *Ncs1* knock-down in the hippocampus impaired displaced object memory (Fig. 4H) while sparing the recognition of novel objects (Fig. 4I). *Ncs1* siRNA-treated mice displayed reduced rearing and hole-board test performance (Fig. 4D,E). Acute reduction of *Ncs1* in the hippocampus also abolished the correlation between exploration and learning (Fig. 4J; Supplementary Table S1), and again, we found no correlation between horizontal movement and spatial memory (Fig. 4K; Supplementary Table S1). These data show that the DG and NCS-1 both underlie the motivation to explore novelty, as well as the associated learning bonus.

## Discussion

Our study establishes that animals, actively influencing their experience of sensory information through intrinsically motivated exploration, activate specific brain structures that promote efficient learning. Furthermore, we identified a molecular basis behind the LM benefit associated with self-directed exploration, demonstrating NCS-1 and the DG are both required. Our findings suggest that the fundamental need to explore, which is present in many species including humans, may be tied to their LM abilities. Exploration is thus a crucial facet of behavior for reasons that go beyond direct material benefits of exploring a given environment.

Novel stimuli can induce different forms of exploratory behavior, chiefly depending on their perceived potential to deliver pain or reward. Fear and novelty-driven exploration are thus inversely related, producing a continuum of behavior that may belie a common neurobiological basis. Indeed, dopaminergic projections to the ventral hippocampus and basolateral amygdala influence motivation and aversive reaction through tonic and phasic components, the effects of which appear to be D2DR-dependent^31^. Consistent with this, our data suggest that dimly-lit environments facilitate exploration by both reducing the “threatening” properties of novel objects, as well as promoting their “rewarding” characteristics. We confirmed that behavior in brightly-lit conditions is associated with changes in anxiety-related behaviors, including reduced rearing, increased grooming activity, and increased avoidance of the center of the arena. The competing balance of survival drives, namely safety vs. exploration and their associated neural circuitries, likely determine an animal’s willingness to engage with its environment. Indeed, these drives likely act in concert to influence LM directly, and in synergy with the animal’s sensory experience to form the basis of contextual memory in an NCS-1-dependent manner. Animals with either acute *Ncs1* knock-down in the DG, or chronic and global NCS-1 reduction, do not demonstrate a self-directed exploration learning bonus, and therefore the novel NCS-1 P144S mice generated and characterized here may represent a powerful animal model for additional mechanistic study.

Using immediate early gene expression, we identified neural circuitry induced by environments that favor exploration. SNS conditions that foster exploration, enhanced *Fos* expression in the DG, CA3 and NAc. In human fMRI studies, the NAc, the main projection target of dopaminergic neurons of the substantia nigra/ventral tegmental area, are activated for reward anticipation^32^. Both novelty and reward cues (as motivational factors) co-activate the substantia nigra/ventral tegmental area and hippocampus^33^,^34^. Interestingly, this is coherent with the impairment observed in the object recognition task after pharmacological manipulation of the NAc and DG in mice^35^,^36^. Specifically, novelty facilitates the induction and persistence of long-term plasticity in the DG^37^,^38^, processes considered cellular mechanisms for LM. Our data suggests that NAc-hippocampal communication modulates the salience of environmental features that in turn promotes fine-tuning of exploratory behavior necessary for LM.

Self-directed exploration is also important clinically. Reduced exploration of novel stimuli is prevalent in memory deficit disorders, including Alzheimer’s disease^39^, fragile X syndrome^40^, age-associated cognitive decline^41^, as well as other learning disabilities including autism^42^. In addition, patients with a number of psychiatric disorders, such as schizophrenia^43^ and depression^44^, exhibit impairments in exploration or LM or both. Therapeutically, finding novel molecular targets, such as NCS-1, to ameliorate cognitive deficits in these disorders may be possible by focusing on the mechanisms coupling self-directed exploration to efficient learning. Some evidence from other candidate genes, notably PDE4B, suggests that this is possible, as genetic inhibition of PDE4B lead to improvements in both exploration and LM, potentially through effects on perceived environmental threat^45^.

Non-threatening educational practices that support information gathering and investigation may be of benefit to student academic success, development of identity, and future adaptation^46^,^47^. In particular, the efficiency of language learning exhibits a reciprocal correlation with environmental stress^48^, and curiosity may be beneficial for life-long learning in nurses^49^. Our data provide evidence that a learning bonus is produced by exploration in non-threatening novel environments and reveal insights into the molecular and anatomical basis for these benefits. Further investigation of the link between novelty-induced exploration and memory may prove fruitful in the search for better therapeutic strategies for cognitive dysfunction associated with aging, neurodegenerative, and neuropsychiatric disorders.

## Materials and Methods

### Behavioral Testing

Mice were housed (five per cage) and tested at the Toronto Centre for Phenogenomics (TCP; Toronto, Canada) in HEPA-purified, temperature- and humidity-controlled rooms with 12–12 h light–dark cycle (lights on at 07:00). Animal use protocols were approved by the local committee on animal care at TCP and conformed to the national guidelines (CCAC; http://www.ccac.ca). Food pellets and water were available ad libitum, except during behavioral testing. Animal handling was done every day starting from 10 days before the behavioral tests. Experiments were performed during the light cycle, from 8:00 AM to 11:00 AM. Experimenters were blind to the genotypes or treatments of the subjects. Most behavioral tests were scored using OBSERVER 5.0 software (Noldus Information Technology, Leesburg, VA, USA). Otherwise, it was automated scoring.

### Discovery of the *Ncs1* ^*P144S/P144S*^ mouse line

We found one mutation in *Ncs1* at residue 144 (Pro to Ser), a region between the third and fourth EF-hands, after searching > 2000 ENU-treated mouse genomic DNA samples, in collaboration with RIKEN in Japan. To limit the effects of unknown point mutations, *Ncs1*^*P144S/P144S*^ mice were backcrossed 10 generations onto C57BL/6J. See Supplementary Information for additional details.

### Open Field Conditions

Handled subjects were placed in an empty clear Plexiglas chamber (42 × 42 × 42 cm; AccuScan Instruments, Columbus, OH, USA). The chamber was lit overhead at the following intensities: Novel Dim or Familiar Dim at 20–40 lux and Novel Bright or Familiar Bright at 400–500 lux. The Familiar Dim and Familiar Bright groups were exposed on the previous day to the same chamber under dim lighting (20–40 lux). Mice were recorded over 30 min and the behaviors were scored by an automated in VersaMax animal activity monitoring system (AccuScan Instruments). Mice were then sacrificed 30 min or 90 min post-exposure to open field for real time PCR (RT-PCR) or c-fos detection, respectively.

### Object Recognition Test

Experiments were conducted in Plexiglas chambers (same as in section 2.2.1). The test consisted of three phases: (1) habituation/training (15 min) with either dim (20–40 lux) or bright lighting (400–500 lux), with four identical objects presented, (2) displaced object phase (5 min) under dim lighting with two objects displaced towards the arena center (Fig. 1A), and (3) novel object discrimination phase (5 min) under dim lighting by presenting one familiar object from the training phase and one novel object (Fig. 1A). Between phases, mice were returned to their home cages for 3 min. In all phases, the positions of the nose, tail and center of mass of each mouse were tracked using EthoVision 7.0 software (Noldus Information Technology) and analyzed the speed of movement and total distance travelled. In addition, time spent on attending to objects and the number of rearings were observed and scored manually using Observer 5.0 software (Noldus information Technology). During habituation/training, in the first 5 min, the number of rearings was scored, and the speed of subject and total distance travelled were analyzed. The criteria used for scoring the behaviors were: The total number of rearings—the sum of unsupported rearings, rearing to the walls and rearing to the objects. Unsupported Rearing—animal is upright and supported exclusively on hind legs, potentially sniffing the environment. Attending to objects—whenever mice were actively investigating the objects, generally by sniffing within 1 cm.

### Hole-board Test

Behavioral observations were made for 5 min in a dimly-lit or a brightly-lit clear Plexiglas chamber of the same size as the one used in object recognition testing, but containing 8 circular holes (r = 1 cm) in the center, two holes at the corner (r = 2 cm) and 2 rectangular holes on the wall (width = 2 cm height = 1 cm) elevated 8 cm above a clean surface. The behavioral activities such as time spent exploring the holes and number of rearings were scored.

### New Frontier Exploration Test

The new frontier exploration test was conducted as described previously^9^. Mice were allowed to climb from their home cage onto any of four platforms, each elevated 15 cm above the floor. The platforms connected the home cage to novel environments (18 cm × 30 cm), also 15 cm above the floor. A subject with two or more paws in the novel environment was recorded as a crossing event. A visit to the same novel platform environment was only counted as a second crossing event if subjects subsequently returned to their home cage area. Mice were given 15 min to explore the platforms.

### Botulinum neurotoxin A (BoNT) Injections

C57BL/6J (8–10 weeks old, TCP, Canada) mice were randomly divided into two groups of 20 mice. Incobotulinumtoxin A (BoNT, Xeomin; Merz Pharma, Canada Ltd., Burlington, ON, Canada) was reconstituted and diluted in PBS. Two dilutions were made, 0.2 LD50 mouse units (MU, or U) and 0.1 U of BoNT per 20 μL. Injections were performed with a Hamilton microsyringe (33G; Hamilton, Reno, NV, USA) with a volume of 20 μL in one or both hindlimb muscles, similar to described work^21^. After injection, the mice were observed daily and tested 3 days after the BoNT injections.

### Animal Surgery and Infusion of small interfering RNA (siRNA)

Male C57BL/6J (10–12 weeks old, TCP) were handled while housed five per cage for at least 1 week before surgery, and subsequent to surgery for hippocampal cannula insertion were housed one per cage. We delivered *in vivo* either *Ncs1* siRNA (Mm Freq 3 HP siRNA; QIAGEN, Valencia, CA, USA) (Supplementary Figure S5) or negative control siRNA (Shanghai GenePharma Ltd., Shanghai, China) as per published methods^47^. For the injection of siRNA (fear memory test), mice were anesthetized with isoflurane and stainless steel guide cannulae (custom built by Small Parts) were cemented onto the skull after positioning the tip at coordinates -2.0 mm from bregma, +/−1.5 mm from midline and −1.2 mm from the dura. Seven days after recovery from surgery, animals were injected with siRNA or peptide. Each siRNA was diluted to 0.5 μg/μL in 5% glucose and mixed with six equivalents of a 22 kDa linear polyethyleneimine (PEI) (Fermentas Inc., Burlington, ON, Canada). After 10 min of incubation at room temperature, 2 μL were injected into each hippocampus through an infusion cannula protruding 0.5 mm below the termination of the guide cannulae (to −1.7 mm from the dura) with a Harvard Precision Pump at 0.5 μL/min for 2 min, for a total of 1 μL per hemisphere. Animals were handled gently to minimize stress. A total of three infusions of siRNA were given over a period of 3 days (1 μg siRNA per hippocampus per day). Mice were trained 3 days after the last siRNA injection and tested 24 h later.

### Immunohistofluorescence; c-Fos staining

Sixty minutes after completing the novelty test session in the open field (Fig. 1), each animal was anaesthetized with isoflurane and transcardially perfused with 20 mL of saline solution followed by 20 mL of 4% paraformaldehyde (PFA) in PBS. Each brain was rapidly removed and post-fixed overnight in 4% paraformaldehyde in PBS. 50 μm coronal sections were obtained using a vibrating microtome (VT10005; Leica Microsystems, Weltzlar, Germany) and stored at −4 °C in PBS. Sections to be processed for c-fos-immunoreactivity were transferred to PBS (pH 7.4) and washed several times. After 1 h of incubation in PBS containing 5% BSA and 0.1% Triton X-100 (PBS-BT), sections were incubated overnight in anti-Fos rabbit polyclonal antibody (sc-52; Santa Cruz Biotechnology, Santa Cruz, CA, USA) diluted 1:500 in PBS-BT, at 4 °C and with constant orbital rotation. Sections were washed three times in PBS and incubated in secondary antibody diluted 1:1000 in PBS-BT (goat anti-rabbit IgG; Cy5-conjugated; Jackson ImmunoResearch, West Grove, PA, USA) for 2 h at room temperature. After several PBS rinses, sections were mounted onto slides using Prolong Gold media (Life Technologies), and cover slipped for microscopical examination. Sections from groups to be directly compared were processed at the same time and using the same conditions in order to reduce variability.

### Analysis of c-Fos Positive cells

In all the experiments, the number of cells displaying c-Fos immunoreactivity was measured in the following brain regions: NAc, the CA1, CA3 and DG sub-regions of the hippocampus, and amygdala. The brain regions were defined using stereotaxic coordinates^48^. At least two to three non-consecutive sections were stained and imaged bilaterally for each brain region, for each subject. Fluorescence images were acquired at 20X (0.75 NA) magnification, using a laser-scanning confocal microscope (Nikon C1si; Nikon Canada, Mississauga, Canada). After image acquisition, counting of the labeled cells was carried out using NIS-Elements AR software (Nikon Canada). Briefly, for each region, positive cells were automatically detected based on their intensity of staining relative to background and their size. The experimenter was blind to experimental grouping throughout image acquisition and processing. Counts from both hemispheres and from all rostro-caudal levels were averaged in order to obtain a single value for each subregion, for each subject. For each experiment, the count of c-Fos-positive cells from each region was normalized by dividing it by area, in order to allow comparison of the relative novelty-induced change between the various brain regions and between different experiments.

### Quantitative real-time polymerase chain reaction (RT-PCR)

Right after completing the novelty test sessions in the open field (Fig. 1), mice were euthanized by cervical dislocation. Hippocampi were dissected in ice-cold PBS for RNA extraction. RNA was isolated using Trizol (Invitrogen^TM^ Life Technologies, Burlington, ON, Canada) according to the manufacturer’s specifications. Complementary DNA was generated using Reverse transcriptase III (Invitrogen^TM^ Life Technologies). Complementary DNA was synthesized and real-time polymerase chain reaction (PCR) performed using ABI prism and SDS 2.1 software. ABI assays on demand (AppliedBiosystems Inc., Foster City, CA, USA) were used for *Ncs1, Drd2, Creb, Fos, Gapdh* and *Actin*. Quantitative PCR were run in triplicate and threshold cycle (Ct) values averaged. Data were then normalized to *Gapdh*. A region of the *Ncs1, Drd2, Creb, Fos, Gapdh* and *Actin* mRNA was amplified using primers shown; NCS1 2F (5′- CTG AAG TTG TGG AGG AGC TG-3′) and NCS1 2R (5′- CTT GTT CTC GTC GAA GAC G-3′), mGAPDH F (5′-GCA CAG TCA AGG CCG AGA A-3′) and mGAPDH R (5′-GCC TTC TCC ATG GTG GTG AA-3′), D2DR F (5′-TAT GCC CTG GGT CGT CTA TC-3′) and D2DR R(5′-AGG ACA GGA CCC AGA CAA TG-3′), CFOS F (5′-CTC CCG TGG TCA CCT GTA CT-3′) and CFOS R (5′-TTG CCT TCT CTG ACT GCT CA-3′), CREB F (5′-CTT CCA CTT CTG CCC TCA AG-3′) and CREB R (5′-TCC CTA AGG CAA TCA TGG AG-3′), mActin F (5′-CGG TTC CGA TGC CCT GAG GCT CTT-3′) and mActin R (5′-CGT CAC ACT TCA TGA TGG AAT TGA-3′).

### Western blotting

Mice were sacrificed by cervical dislocation, the hippocampi removed quickly and frozen on dry ice. Each hippocampus was lysed in 300 μL radioimmunoprecipitation assay (RIPA) buffer (Santa Cruz) containing Roche complete protease inhibitor tablet. Protein concentrations were determined using Bio-Rad Bradford protein assay kit (Bio-Rad Laboratories) according to the manufacturers’ instructions. 40 μg of lysate was separated by sodium dodecyl sulphate-polyacrylamide gel electrophoresis (SDS-PAGE) and blotted onto nitrocellulose membranes. Immunodetection of proteins was performed according to standard procedures using polyclonal primary antibodies against NCS-1 (ProteinTech Group or Abcam) and anti-rabbit IgG (HRP-conjugated; GE Healthcare). For each anatomical region, NCS-1 protein levels were normalized to β-tubulin on the same blot.

### Statistical analyses

Behavioral data were analyzed by mixed factor analysis of variance (ANOVA) or repeated measures of ANOVA, followed by a Bonferroni’s post-hoc testing. Elsewhere, two-tailed *t* tests for statistical significance were employed. Values in figures are expressed as mean ± standard error of the mean (SEM). Correlation data were analyzed with a Spearman correlation test. Differences were considered statistically significant at p < 0.05.

## Additional Information

**How to cite this article**: Mun, H.-s. *et al.* Self-directed exploration provides a *Ncs1*-dependent learning bonus. *Sci. Rep.* **5**, 17697; doi: 10.1038/srep17697 (2015).

## Supplementary Material

Supplementary Information
