# Supplementary material for: Self-directed exploration provides a Ncs1-dependent learning bonus
Source: Sci Rep. 2015 Dec 7;5:17697. doi: 10.1038/srep17697 (PMC4671055; doi:10.1038/srep17697)
Supplement: Supplementary Information [file srep17697-s1.pdf]

Supplementary Information

Self-directed exploration provides a NCS-1 dependent learning bonus

Ho-suk Mun, Bechara J. Saab[†], Enoch Ng, Alexander McGirr, Tatiana V. Lipina, Yoichi Gondo, John Georgiou[†], and John C. Roder

[†]To whom correspondence should be addressed at:
John Georgiou; Lunenfeld-Tanenbaum Research Institute, Mount Sinai Hospital, 600 University Avenue, Toronto, ON, Canada, M5G 1X5; E-mail: georgiou@lunenfeld.ca
Bechara J. Saab; Preclinical Laboratory for Translational Research into Affective Disorders, University of Zurich, Zurich CH-8008, Switzerland; E-mail: bechara.saab@uzh.ch

Inventory of Supplementary Information:

Supplementary Information includes Supplementary Experimental Procedures, 1 Supplementary Table, 5 Supplementary Figures and Supplementary References.

1. Supplementary Experimental Procedures: including (1) Discovery of the $Ncs1^{P144S/P144S}$ Mouseline and Genotyping**,** (2) Elevated Plus Maze, (3) Accelerating Rotarod, (4) Forced Swim Test, (5) Sucrose Preference Test, and (6) Transfection of siRNA into N2A cells.

2. Supplementary Table 1: Statistical summary of correlation analysis.

3. **Supplementary Figure 1**: Related to main Figure 1. Behavioral activity in the open field test and object recognition test in the novel dim, novel bright or familiar dim conditions as well as BoNT-treated group compared to saline group.

4. Supplementary Figure 2: Related to main Figure 2. Confocal images of c-Fos immunohistofluorescence.

5. Supplementary Figure 3: Related to main Figure 3. DNA sequence and chromatogram of $Ncs1^{+/+}$ and $Ncs1^{P144S/+}$ and reduced NCS-1 protein levels in hippocampus but no changes in body weight, motor function and performance for anxiety or depression of $Ncs1^{P144S/P144S}$ mice.

6. Supplementary Figure 4: related to main Figure 3. Behavioral activity in the open field and hole poke tests, no **object bias in the object recognition test for $Ncs1^{P144S/P144S}$ mice.**

7. Supplementary Figure 5: related to main Figure 4, NCS-1 levels after knockdown in N2A cells and behavioral activity in the first 5 min of open field test.

8. Supplementary References

Supplementary Experimental Procedures

Discovery of the *Ncs1*$^{P144S/P144S}$ ***Mouseline and Genotyping.*** In collaboration with RIKEN, one mutation in *Ncs1* was found after screening over 2,000 mouse genomic DNA samples of DBA/2XC57BL/6F1 males (= G1 males) derived from ENU-treated C57BL/6 males (=G0 males). The C to T mutation in *Ncs1* is located within exon 6 at position 2:31,284,698 (ENSMUSG00000062661) and changes the proline at position 144 to a serine (P144S) in a region between the third and fourth EF-hand (Fig. 3A; S3A, B). Note that there are no known SNPs between C57BL/6J and DBA at or near the mutation site. Once the P144S mutation in NCS-1 was identified, the sperm were used to rederive mutant mice (*Ncs1*$^{P144S/+}$) by *in vitro* fertilization and implantation into a pseudopregnant C57BL/6J female. Therefore, the mice obtained from this process were only 2nd generation C57BL/6J and contained several mutations (as many as 30 non-silent point mutations in coding regions of genes) in addition to contaminating donor DBA alleles. To limit the chances that resulting phenotypes were due to unknown point mutations or DBA genes, we followed the standard procedure that was to backcross for 10 generations. The NCS (P144S) mouseline (RBRC-GD003518) is available from RIKEN BioResource Center (http://www.brc.riken.jp/lab/animal/en/)."

Genotyping of mice was performed by PCR using DNA from tail clips and the following primers to amplify *Ncs1*; forward primer, 5'-GCC TAA GGC TGC ACT GAC TC-3'; Reverse primer, 5'-TGG GAA ACT CAC CAA AGA CC-3', which yielded a PCR product of 245 bp. The PCR product of the *Ncs1 P144S* mutant allele was cut by Hpy188I (New England Biolabs, Beverly, MA, USA), such that the wild-type allele ran as a 245 bp product while the resulted products were 245, 168 and 84 bp fragments for *Ncs1*$^{P144S/+}$ and 168 and 84 bp fragments for *Ncs1*$^{P144S/P144S}$.

Elevated Plus Maze. The elevated plus-maze test was conducted as previously described (1). The apparatus consisted of two open arms (25 × 5 cm; 70 lux) and two closed arms (25 × 5 × 30 cm; 1.3 lux) extending from a central platform (5 × 5 cm) and elevated 50 cm from the ground. The floor of the arms was made of white Plexiglas and the walls of the closed arms were made of black Plexiglas. Similar arms were opposite to each other and at a 90° angle from dissimilar arms. The test mouse was placed in the central area facing an open arm and allowed to explore the apparatus for 5 min. The number of entries and head dips, and the time spent in the open arms, closed arms, and central platform were recorded.

Accelerating Rotarod. This experiment was performed as previously described (1) using an Economex Rotarod apparatus (Columbus Instruments, Columbus, OH, USA). The original 3 cm ribbed plastic rotating axle was divided using four adjustable flanges, which enabled testing a maximum of four mice simultaneously. The rod was suspended at a height of 30 cm above the plastic surface. Mice were placed on top of the rod, facing away from the experimenter. In this orientation, forward locomotion opposite to rotation of the rod is necessary to avoid falling. During the stationary mode, each mouse was first observed for 10 seconds without any rotation. The axle was then adjusted for a constant speed of 5 r.p.m., and each mouse observed for 10 seconds (fixed speed mode). Next, beginning at 5 r.p.m., the rotation gradually increased in increments of 0.1 r.p.m. per second and the latency to fall off the axle was recorded in seconds for each mouse for the maximum period of 300 seconds (accelerating speed mode). The mean latency was then calculated by averaging the latency for three consecutive trials. The stationary and fixed speed mode sessions were training periods and allowed the animals to become accustomed to the apparatus. The accelerating Rotarod procedure was repeated for 3 consecutive days at 3 trials per day to measure performance and motor learning.

Forced Swim Test. The forced swim depression protocol was performed as described (2). The mice were released individually into a transparent plastic cylinder (25 cm height, 18 cm diameter), which contained water at 25°C to the depth of 18 cm. The experiment lasted 6 min, and an observer scored the following parameters in the last 4 min of the trial: (i) active swimming (including crossing the quadrants of the container) and (ii) floating (no limb movement and making only minimal movements to keep the head above the water). Each mouse was allowed to dry after the test, and the water was changed between subjects.

Sucrose Preference Test. A 5-day sucrose preference protocol was conducted as described (3). Mice were individually housed and were presented with two identical water bottles with balled stoppers. The positions of the bottles were alternated daily to avoid a side bias. The weight of each water bottle was recorded daily to assess the amount of solution consumed. On days 1 and 2 mice were presented with two identical bottles filled with water (water/water). On days 3 and 4 bottles contained 5% sucrose solution dissolved in the drinking water (sucrose/sucrose). On day 5 one bottle was filled with water and the other was filled with 1% sucrose solution. Preference for sucrose was calculated as 100 x

sucrose/(sucrose+water) consumption.

Transfection of siRNA into N2A cells. The mouse neuroblastoma cells (N2A; ATCC, Rockville, Maryland, USA: CCL 131) cells were maintained in Dulbecco's modified Eagle's medium (Invitrogen™ Life Technologies, Carlsbad, CA, USA) supplemented with 10% cosmic calf serum (HyClone) and 2 mM L-glutamine. All of the cell cultures were maintained at 37°C in 5% CO_2. Double-stranded siRNA was transfected into N2A cells using RNAimax (Invitrogen™ Life Technologies) as the transfection reagent. Before transfection, the cells were washed and resuspended in 900 μL of RPMI. Cationic lipid complexes, prepared by incubating 2 μM siRNA duplexes with 3 μL of oligofectamine in 100 μL of RPMI medium, were added to the wells. After a 24 hours transfection, the NCS-1 level was measured by western blot.

	Comparison	Slope	Pearson r	r^2	F-value	P-value
**Novel Dim (n=14)**	**Rearing-DO**	**0.72 ± 0.26**	**0.63**	**0.39**	**F(1,12)=7.8**	*****
	Rearing-NO	-0.21 ± 0.36	-0.17	0.03	F(1,12)=0.34	0.57
	Horizontal-DO	-0.22 ± 0.17	-0.04	0.00	F(1,12)=0.02	0.90
	Horizontal-NO	-0.11 ± 0.18	-0.18	0.03	F(1,12)=0.38	0.55
**Novel Bright (n=15)**	Rearing-DO	0.29 ± 0.21	0.35	0.12	F(1,13)=1.8	0.21
	Rearing-NO	0.61 ± 0.33	0.45	0.20	F(1,13)=3.3	0.09
	Horizontal-DO	0.05 ± 0.13	0.10	0.01	F(1,13)=0.14	0.71
	Horizontal-NO	-0.20 ± 0.30	-0.15	0.03	F(1,13)=0.44	0.52
**Uni hindlimb Saline injection (n=10)**	**Rearing-DO**	**0.49 ± 0.06**	**0.94**	**0.89**	**F(1,8)=62**	*******
	Rearing-NO	0.39 ± 0.33	0.39	0.15	F(1,8)=1.4	0.27
	Horizontal-DO	-0.05 ± 0.12	-0.14	0.02	F(1,8)=0.17	0.69
	Horizontal-NO	0.14 ± 0.23	0.22	0.05	F(1,8)=0.39	0.55
**Uni hindlimb BoNT injection (n=10)**	Rearing-DO	0.70 ± 0.37	0.56	0.31	F(1,8)=3.60	0.09
	Rearing-NO	0.05 ± 0.81	0.02	0.00	F(1,8)=0.00	0.95
	Horizontal-DO	0.11 ± 0.18	0.20	0.04	F(1,8)=0.35	0.57
	Horizontal-NO	-0.46 ± 0.30	-0.48	0.23	F(1,8)=2.3	0.16
**Bi HPC injection Negative siRNA (n=16)**	**Rearing-DO**	**0.45 ± 0.11**	**0.74**	**0.55**	**F(1,14)=17**	*******
	Rearing-NO	0.18 ± 0.13	0.34	0.11	F(1,14)=1.8	0.20
	Horizontal-DO	-0.03 ± 0.18	-0.05	0.00	F(1,14)=0.03	0.87
	Horizontal-NO	0.06 ± 0.16	0.09	0.01	F(1,14)=0.13	0.73
**Bi HPC injection *Ncs1* siRNA (n=16)**	Rearing-DO	0.07 ± 0.19	0.10	0.01	F(1,14)=0.14	0.72
	Rearing-NO	-0.31 ± 0.22	-0.35	0.12	F(1,14)=1.9	0.19
	Horizontal-DO	0.19 ± 0.14	0.33	0.11	F(1,14)=1.7	0.21
	Horizontal-NO	-0.23 ± 0.16	-0.35	0.13	F(1,14)=2.0	0.18
***Ncs1*$^{+/+}$ (n=16)**	**Rearing-DO**	**0.95 ± 0.30**	**0.64**	**0.41**	**F(1,14)=9.8**	******
	Rearing-NO	0.47 ± 0.28	0.41	0.17	F(1,14)=2.8	0.12
	Horizontal-DO	-0.15 ± 0.12	-0.31	0.10	F(1,14)=1.5	0.24
	Horizontal-NO	-0.05 ± 0.10	-0.15	0.02	F(1,14)=0.32	0.58
***Ncs1*$^{P144S/P144S}$ (n=15)**	Rearing-DO	0.48 ± 0.44	0.29	0.08	F(1,13)=1.2	0.30
	Rearing-NO	0.55 ± 0.33	0.42	0.17	F(1,13)=2.7	0.12
	Horizontal-DO	0.03 ± 0.18	0.04	0.00	F(1,13)=0.02	0.89
	Horizontal-NO	-0.14 ± 0.13	-0.28	0.08	F(1,13)=1.1	0.31

Supplementary Table 1. Statistical summary of correlation analysis. The analysis of correlation between explorational activity (Rearing or Horizontal activity) and preference to objects (Novel or Displaced objects, NO and DO respectively) was performed and slope, Pearson r, r^2, F-value and P-value are shown for all the conditions related to main Figure 1, 3 and 4. * P = 0.05, ** P = 0.01, *** P < 0.001.

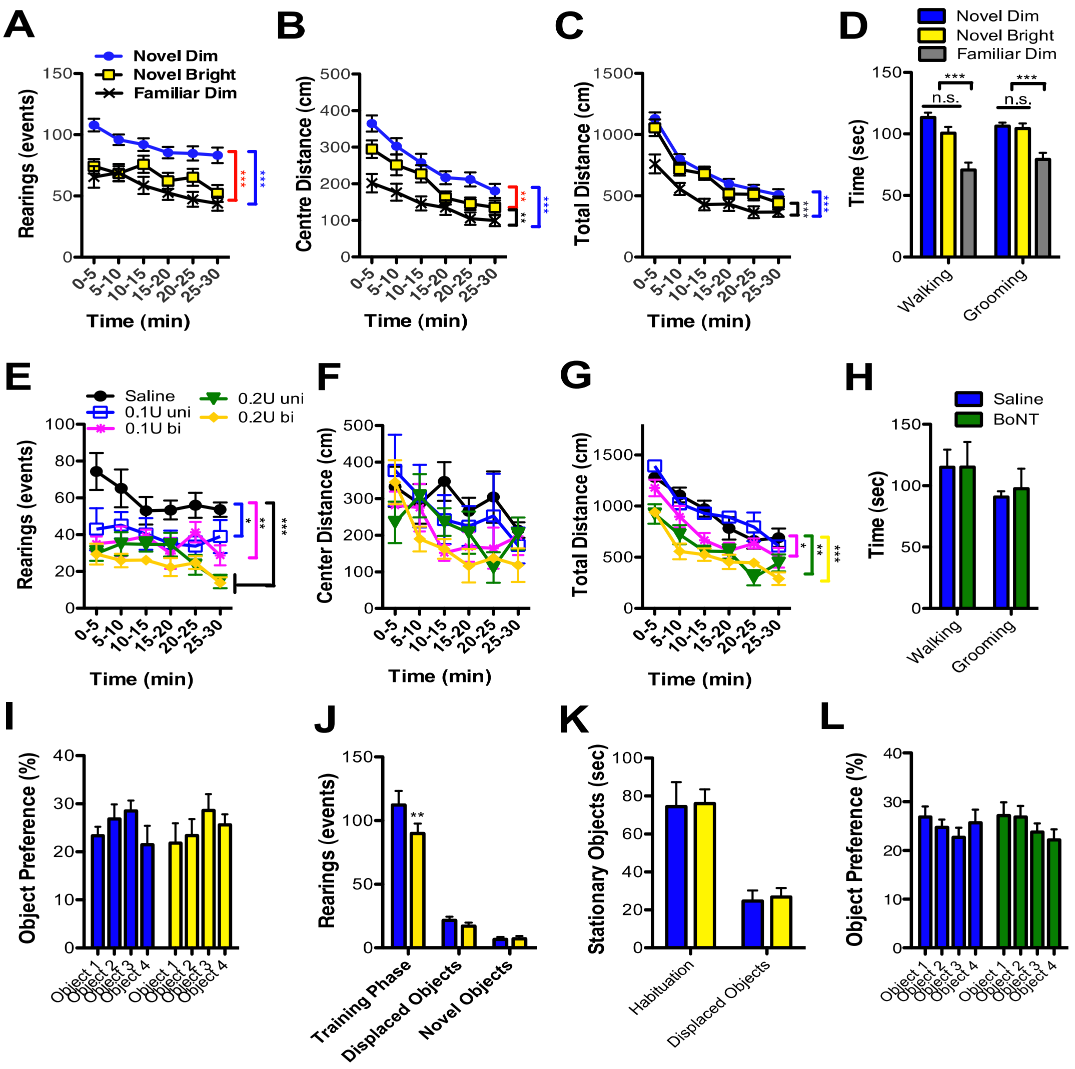

Supplementary Figure 1. Related to main Figure 1. Behavioral activity in the open field test and in the object recognition test in the novel dim, novel bright or familiar dim conditions as well as BoNT-treated group compared to saline-treated group.

(A-D) In the open field, C57BL/6J mice were tested in novel dim (n=35), novel bright (n=31) or familiar dim (n=29) environments. Graphs show vertical rearing **(A)**, horizontal movement in the centre **(B)** or in the entire arena (taken as a measure of overall ambulation) **(C)** or walking and grooming behavior in the first 5 min of open field test **(D).** **(E-H)** Comparison of unilateral (uni) and bilateral (bi) botulinum neurotoxin A (BoNT, XEOMIN) hindlimb injections at 0.1 and 0.2U delivery of BoNT injection and testing in the open field three days later (n=10 per group). The delivery of 0.1U uni of BoNT affected the number of rearings **(E)** without differences in ambulation in the center **(F)** or in the entire arena **(G),** or walking and grooming behavior in the first 5 min of open field test **(H)**. **(I-K)** During the object recognition test, **(I)** mice showed no significant preference for any of the four (identical)

objects during the habituation/training session, across all experimental conditions including exposure to bright (n=15) or dim lighting (n=14); **(J-K)** to confirm that mice trained under bright lighting did not interpret the environment during the training phase of the object recognition test as novel, mice trained under dim or bright lighting were assessed for **(J)** the number of rearings in each phase and **(K)** contact time with the two stationary objects during the habituation/training phase and the displaced objects preference phase which showed no differences. **(L)** During the object recognition test**,** mice showed no significant preference for any of the four (identical) objects during the habituation/training session following hindlimb muscle injection with 0.1 U BoNT as compared to saline. Data are expressed as mean ± SEM. *p<0.05, ** P < 0.01, *** P < 0.001.

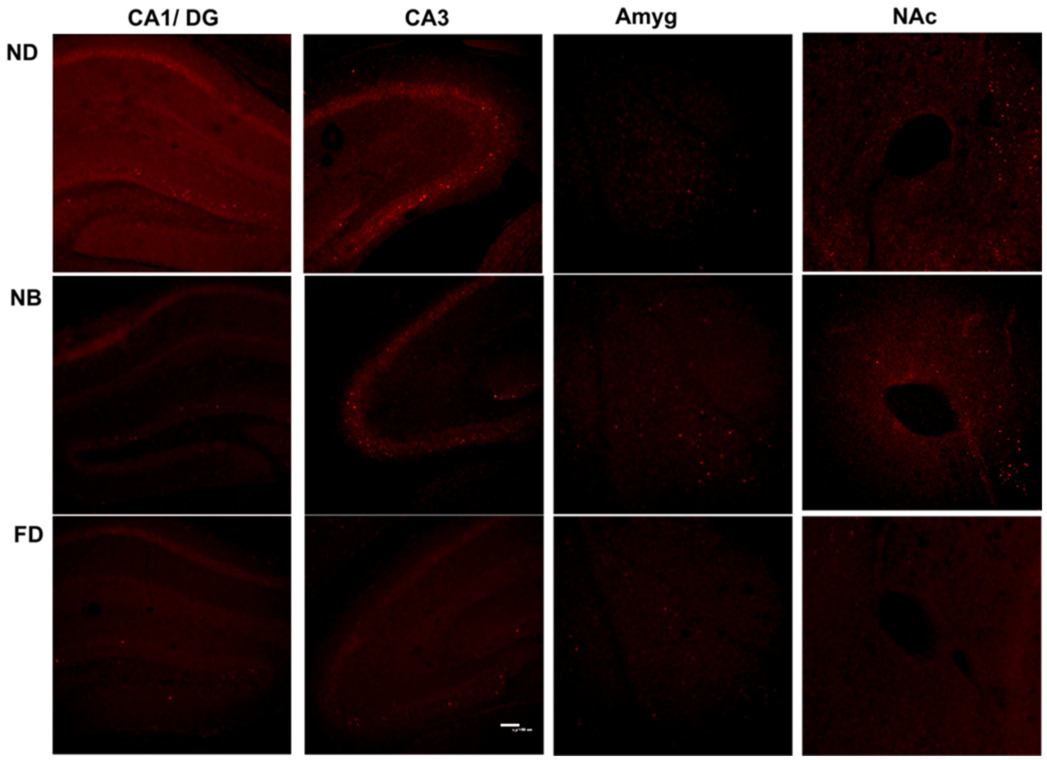

__Supplementary Figure 2__. **Related to main Figure 2. Confocal images of c-Fos immunohistofluorescence.** Sample images showing localization of c-Fos positive cells two hours after exposure to Novel Dim (ND), Novel Bright (NB) or Familiar Dim (FD) environments. Brain regions evaluated include hippocampus CA1/dentate gyrus (DG), CA3, Amygdala (Amyg) and Nucleus accumbens (NAc) (n=3 for each condition). Scale bar = 100 microns.

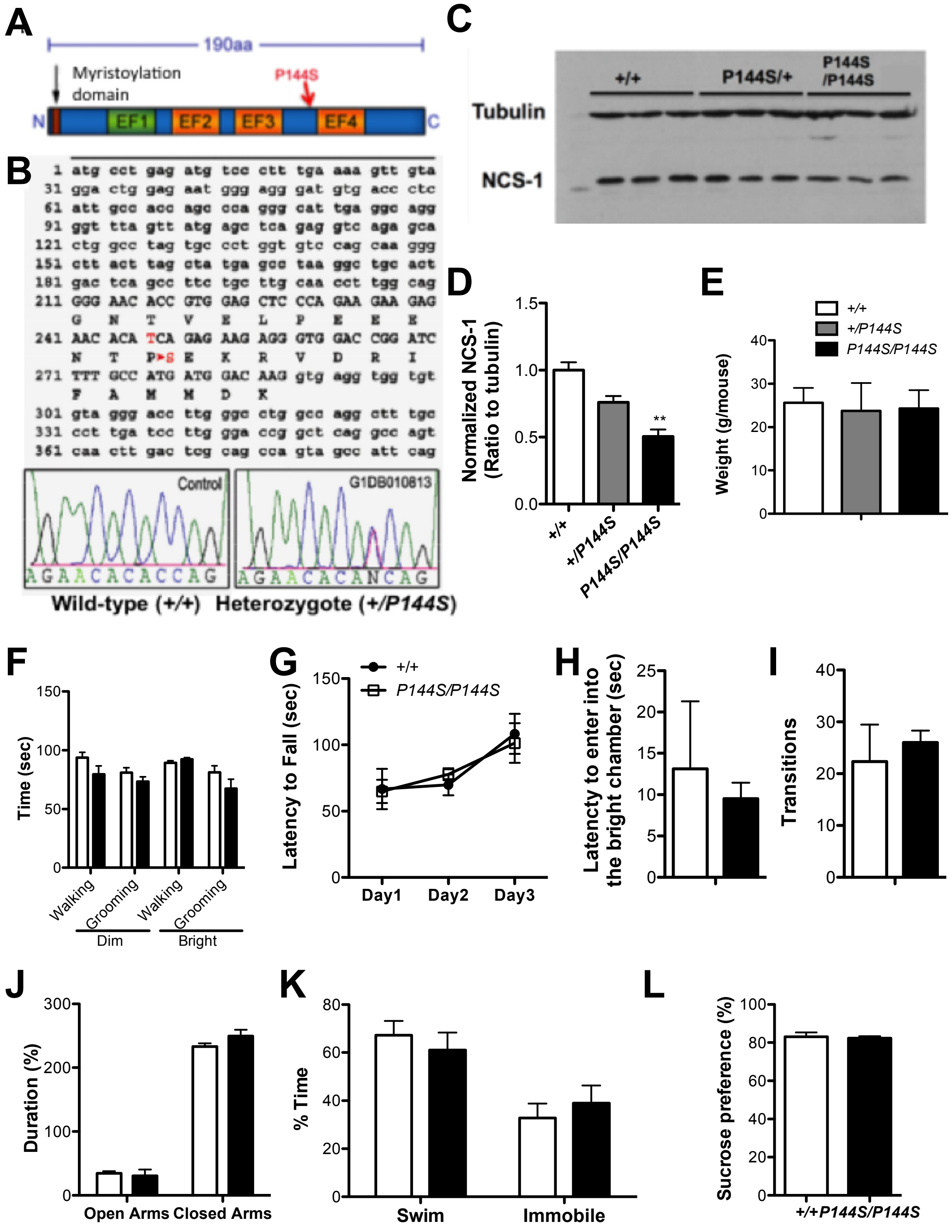

Supplementary Figure 3. Related to main Figure 3. DNA sequence and chromatogram of *Ncs1*$^{+/+}$ and *Ncs1*$^{P144S/+}$ and reduced NCS-1 protein levels in hippocampus but no changes in body weight, motor function and performance for anxiety or depression of *Ncs1*$^{P144S/P144S}$ mice. (A) Diagram showing NCS-1 protein with its structural domains

including EF hands (orange=functional, green=nonfunctional) and the position of the P144S mutation. **(B)** In the upper panel, the 78 bases shown in uppercase letters reflect exon 6 of *Ncs1* and the corresponding amino acid coding is shown underneath. The C to T transition causes a P144S missense mutation (shown in red). In the lower panel, sample sequence chromatograms are shown for a wild-type showing as C and littermate heterozygous mutant showing as N, which signifies mix of C/T. **(C)** Immunoblot of NCS-1 protein (lower bands) from hippocampal lysates of wildtype, P144S heterozygous, and homozygous mice as indicated. A different antibody was used to detect NCS-1 (Abcam) from that employed in Fig. 3C and Fig. 4A (ProteinTech Group). Beta-tubulin (upper bands) was probed simultaneously and levels were used for normalization (n=3 per group). **(D)** Bar plot showing densitometric quantification of normalized NCS-1 levels by genotypes. **(E)** 8 week-old mice were fed with normal chow *ad libitum* and no body weight difference was found between genotypes ($Ncs1^{+/+}$, n=10; $Ncs1^{+/P144S}$, n=16; $Ncs1^{P144S/P144S}$, n=8). **(F)** In the open field, in the first 5 minutes, regardless of lighting conditions, no differences were found in walking and grooming between $Ncs1^{P144S/P144S}$ mutants and littermate controls. **(G)** The rotarod test showed no genotypic differences in motor performance or motor learning (slope) ($Ncs1^{+/+}$, n=6; $Ncs1^{P144S/P144S}$, n=6). **(H, I)** In the Light/Dark box test, no genotypic differences were found in **(H)** latency to enter the brightly-lit chamber and **(I)** number of transitions between the two sides ($Ncs1^{+/+}$, n=6; $Ncs1^{P144S/P144S}$, n=6). **(J)** In the elevated plus maze, the duration spent in the open and closed arms was similar between genotypes ($Ncs1^{+/+}$, n=11; $Ncs1^{P144S/P144S}$, n=8). **(K)** No differences between genotypes in time spent swimming or immobile in the forced swim test ($Ncs1^{+/+}$, n=13; $Ncs1^{P144S/P144S}$, n=12). **(L)** No genotypic differences were found in the amount of sucrose solution (1%) ingested in the sucrose preference test ($Ncs1^{+/+}$, n=6; $Ncs1^{P144S/P144S}$, n=6). Data are expressed as mean ± SEM. $^{**}p<0.01$.

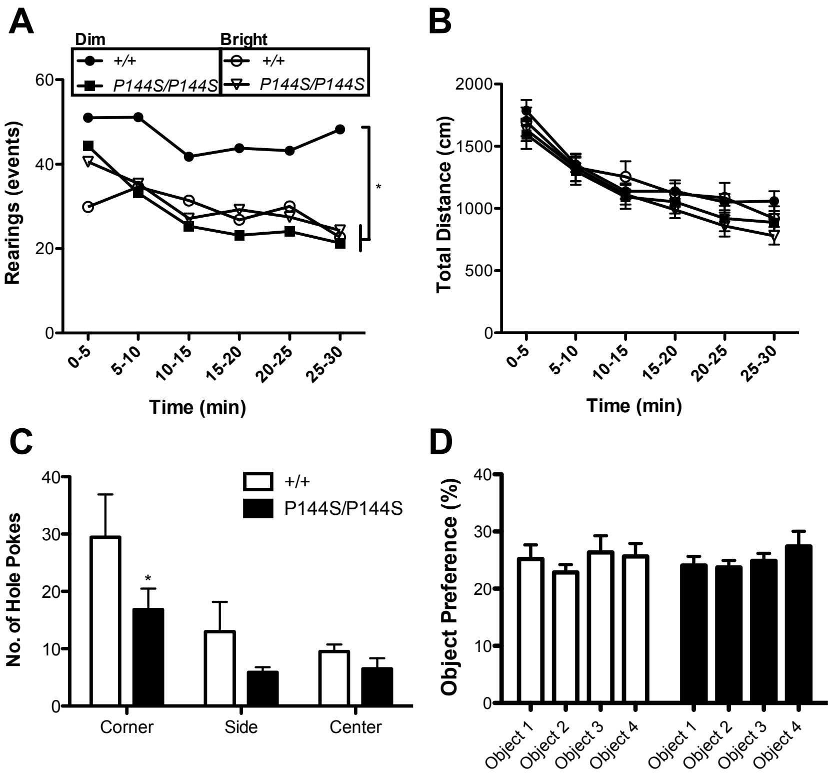

Supplementary Figure 4. Related to main Figure 3. Behavioral activity in the open field test, hole poke test and no object bias in the object recognition test for *Ncs1*$^{P144S/P144S}$ mice. **(A-B)** In the open field test, habituation rate for *Ncs1*$^{P144S/P144S}$ mice under dim and bright lighting. **(A)** Reduced rearing activity in *Ncs1*$^{P144S/P144S}$ mutant compared to wild-type mice (+/+) in the dimly-lit environment. **(B)** There were no statistically significant differences in horizontal activity irrespective of lighting or genotype (*Ncs1*$^{+/+}$ Dim, n=13; *Ncs1*$^{P144S/P144S}$ Dim, n=13; *Ncs1*$^{+/+}$ Bright, n=20; *Ncs1*$^{P144S/P144S}$ Dim, n=20). **(C)** Hole-poke test was conducted and number of hole-pokes in the corners was reduced in *Ncs1*$^{P144S/P144S}$ mice under dim lighting (*Ncs1*$^{+/+}$, n=14; *Ncs1*$^{P144S/P144S}$, n=11). **(D)** No bias towards objects during habituation in the object recognition task was found. Mice showed no significant preference for any of the four identical objects during habituation/training session, across all experimental conditions *Ncs1*$^{P144S/P144S}$ (n=16) compared to *Ncs1*$^{+/+}$ (n=15). Data are expressed as mean ± SEM. *$p<0.05$.

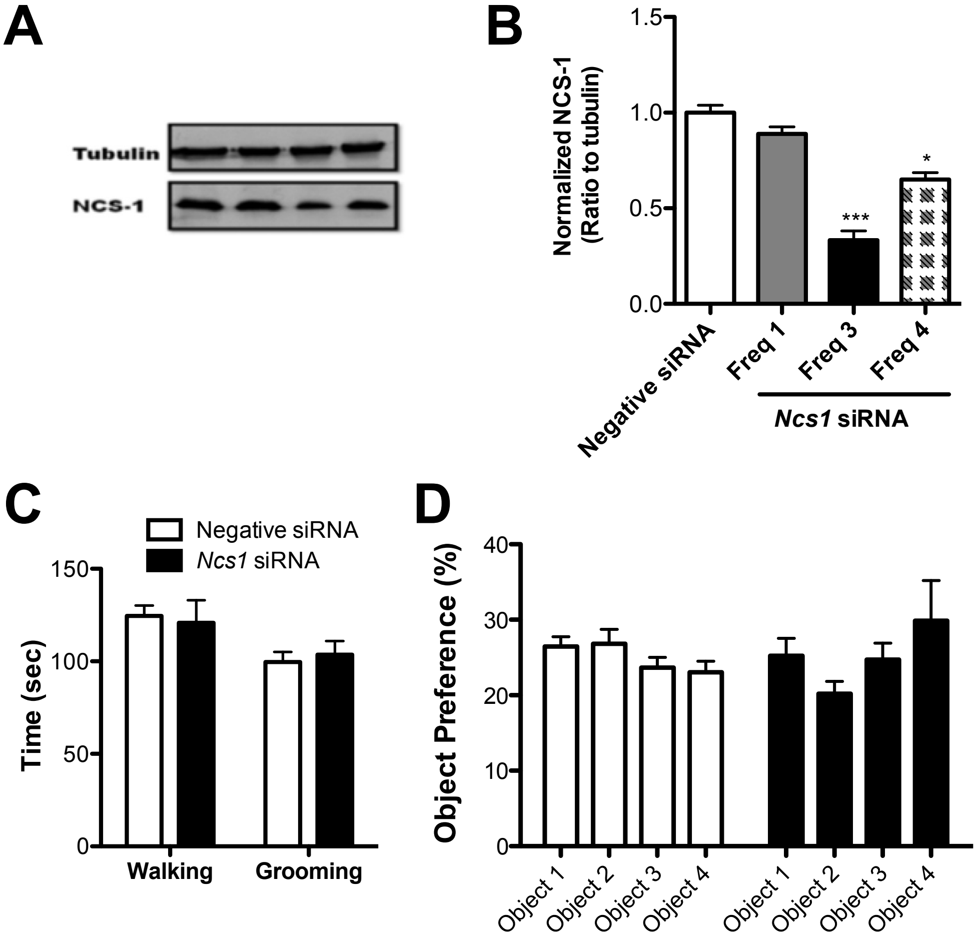

Supplementary Figure 5. Related to main Figure 4. NCS-1 levels after knockdown in N2A cells and behavioral activity in the first 5 min of open field test. (A) Representative immunoblot for NCS-1 and tubulin expression 1 day after siRNA transfection into N2a cell lines. Lane sequence order is as per section **(B)**. **(B)** A graph of quantified relative NCS-1 protein levels in N2A cells after transfection of three different siRNAs against *Ncs1*. Data expressed as a fraction of non-transfected cell NCS-1 levels normalized to tubulin. Compared to Negative siRNA, the Freq 3 siRNA showed the best reduction (67%), while Freq1 siRNA showed minimal reduction and Freq4 siRNA showed 35% reduction of NCS-1 protein level by immunoblot. Freq3 siRNA was chosen for further in vivo experiments. Statistical significance was determined by t-test following densitometric analysis of n=3 blots for each group. **(C)** For behavioral activity in the first 5 min of open field testing**,** no differences were found in walking and grooming between mice infused with *Ncs1* siRNA (n=11) or Negative siRNA (n=11) into the dentate gyrus. **(D)** During the object recognition test, mice showed no

statistically significant preference for any of the four (identical) objects during the habituation/training session, across all experimental conditions of dentate gyrus *Ncs1* siRNA (n=16) or Negative siRNA (n=16) injection. Data are expressed as mean ± SEM. Pearson correlation was used. *$p<0.05$, *** $P < 0.001$.

Supplementary References

1. Soleimani L., Roder J.C., Dennis J.W., & Lipina T. Beta N-acetylglucosaminyltransferase V (Mgat5) deficiency reduces the depression-like phenotype in mice. *Genes Brain Behav* **7**, 334-343 (2008).
2. Cryan J.F., Valentino R.J., & Lucki I. Assessing substrates underlying the behavioral effects of antidepressants using the modified rat forced swimming test. *Neurosci Biobehav Rev* **29,** 547-569 (2005).
3. Roybal K.*, et al.* Mania-like behavior induced by disruption of CLOCK. *Proc Natl Acad Sci U S A* **104,** 6406-6411 (2007).
